# Salicylic acid treatment and overexpression of a novel polyamine transporter gene for astaxanthin production in *Phaffia rhodozyma*

**DOI:** 10.3389/fbioe.2023.1282315

**Published:** 2023-10-19

**Authors:** Jianping Jia, Feifei Li, Yifei Luan, Siru Liu, Zhitao Chen, Guoliang Bao

**Affiliations:** ^1^ School of Public Health, Hangzhou Medical College, Hangzhou, Zhejiang, China; ^2^ Key Laboratory of Drug Safety Evaluation and Research of Zhejiang Province, Hangzhou Medical College, Hangzhou, Zhejiang, China

**Keywords:** *Phaffia rhodozyma*, astaxanthin, phytohormone, antioxidant, signal transduction, polyamine transporter, detoxification, RNA-seq

## Abstract

*Phaffia rhodozyma* represents an excellent microbial resource for astaxanthin production. However, the yeast’s low astaxanthin productivity poses challenges in scaling up industrial production. Although *P*. *rhodozyma* originates from plant material, and phytohormones have demonstrated their effectiveness in stimulating microbial production, there has been limited research on the effects and mechanisms of phytohormones on astaxanthin biosynthesis in *P*. *rhodozyma*. In this study, the addition of exogenous salicylic acid (SA) at a concentration as low as 0.5 mg/L significantly enhanced biomass, astaxanthin content, and yield by 20.8%, 95.8% and 135.3% in *P*. *rhodozyma*, respectively. Moreover, transcriptomic analysis showed that SA had discernible impact on the gene expression profile of *P. rhodozyma* cells. Differentially expressed genes (DEGs) in *P. rhodozyma* cells between the SA-treated and SA-free groups were identified. These genes played crucial roles in various aspects of astaxanthin and its competitive metabolites synthesis, material supply, biomolecule metabolite and transportation, anti-stress response, and global signal transductions. This study proposes a regulatory mechanism for astaxanthin synthesis induced by SA, encompassing the perception and transduction of SA signal, transcription factor-mediated gene expression regulation, and cellular stress responses to SA. Notably, the polyamine transporter gene (PT), identified as an upregulated DEG, was overexpressed in *P. rhodozyma* to obtain the transformant Prh-PT-006. The biomass, astaxanthin content and yield in this engineered strain could reach 6.6 g/L, 0.35 mg/g DCW and 2.3 mg/L, 24.5%, 143.1% and 199.0% higher than the wild strain at the SA-free condition, respectively. These findings provide valuable insights into potential targets for genetic engineering aimed at achieving high astaxanthin yields, and such advancements hold promise for expediting the industrialization of microbial astaxanthin production.

## 1 Introduction

Astaxanthin, chemically known as 3,3′- dihydroxy- β, β′- carotene-4,4′- dione, is a keto-type of carotenoid renowned for its exceptional antioxidant properties. It effectively combats free radicals by reacting with them and neutralizing them using its hydroxyl ketone groups. Astaxanthin’s antioxidant capacity surpasses that of vitamin E by over 500 times (Naguib, 2000; Gao et al., 2012). Furthermore, astaxanthin boasts a wide array of beneficial attributes, including its potential as an anticancer agent, immune system enhancer, liver protector, colorant, and so on. As a result, it finds extensive applications in nutraceuticals, pharmaceuticals, health products, cosmetics, and aquaculture (Hussein et al., 2006; Rodríguez-Sáiz et al., 2010). The primary methods for astaxanthin production encompass extraction from natural sources, chemical synthesis, and biosynthesis (Li et al., 2023). Within the realm of biosynthesis, two key isomers, 3S, 3S′ and 3R, 3R′, have emerged as prominent options due to their superior antioxidant activity, stability, and bioavailability compared to synthetic alternatives. This preference for biosynthetic astaxanthin is projected to result in a 55% reduction in the synthetic astaxanthin market by 2024 (Nutakor et al., 2022).

*Phaffia rhodozyma*, also known as *Xanthophyllomyces dendrorhous*, is a basidiomycetous yeast renowned for its ability to produce astaxanthin as its primary carotenoid. This yeast can utilize various sugars as the carbon source, grow rapidly with a short cultivation cycle, has easier culture conditions, realizes a high-density fermentation, needs less production space, and is generally recognized as safe (GRAS). Moreover, the isomer of astaxanthin produced by *P. rhodozyma* is the 3R, 3R′, which is digested more easily than the 3S, 3S′ isomer, indicating that this yeast is an ideal astaxanthin bioresource (Ramirez et al., 2000; Igreja et al., 2021). However, the astaxanthin productivity in *P. rhodozyma* is still low, which inhibits the industrial production of astaxanthin by *P. rhodozyma* even though by microorganisms. In *P. rhodozyma*, β-carotene is first synthesized through the mevalonate (MVA) pathway, and the β-carotene is then converted to astaxanthin by oxidation reactions. In the MVA pathway, acetyl-CoA is converted to isopentenyl-5-pyrophosphate (IPP) and dimethylallyl pyrophosphoric acid (DMAPP) through 7-step reaction catalyzed by acetoacetyl-CoA transferase (ACAT), 3-hydroxyl-3-methylglutaryl-CoA (HMG-CoA) synthase, HMG-CoA reductase, mevalonate kinase (MK), phosphomevalonate kinase (PMK), diphosphate decarboxylase (PMD) and isopentenyl diphosphate isomerase (IDI). Then, IPP and DMAPP are converted to β-carotene through 4-step of condensation, cyclization and modification catalyzed by geranyl pyrophosphate (GGPP) synthase (crtE), phytoene synthase (crtYB), phytoene desaturase (crtI) and lycopene cyclase (crtYB), in sequence. Finally, β-carotene is converted to astaxanthin through a oxidation reaction catalyzed by astaxanthin synthase (crtS) (Kizer et al., 2008). Acetyl-CoA, NADPH, ATP, Mg ^2+^ as the synthetic materials and cofactors, are essential in astaxanthin synthesis of *P. rhodozyma* (Basiony et al., 2022). Although the genetic transformation system was constructed in *P. rhodozyma* nearly 30 years ago (Adrio and Veiga, 1995), genetic modification of *P. rhodozyma* is still mainly focused on astaxanthin synthesis pathway. For example, *crtE* is a rate-limiting enzyme of astaxanthin synthesis and its overexpression increases the astaxanthin content in *P. rhodozyma* by 1.65-fold compared with the control (Hara et al., 2014a). Three key enzyme genes of the MVA synthetic pathway (*acaT*/*hmgS*/*hmgR*) combined with the *crtE* and *crtS* genes were overexpressed, and the astaxanthin yield of the transformant was 2.1-fold higher than the parent strain without reducing the biomass (Hara et al., 2014b). The global regulation mechanism of astaxanthin synthesis in *P. rhodozyma* is unclear, and the potential targets of genetic modification for astaxanthin improvement are still lack.

In addition, exogenous substances, such as extracts from other organisms, chemical additives, *etc.*, were used to stimulate astaxanthin production in *P. rhodozyma* (Zhang et al., 2020). *P. rhodozyma* was first isolated from plant materials and was proposed to have evolved to adapt the metabolites from its symbiotic plant (Bellora et al., 2016). Thus, the effects of different plant extracts on growth and astaxanthin production in *P. rhodozyma* were analyzed. The Oak leaf extract (at 5% v/v) could increase both astaxanthin content and astaxanthin yield by 1.9-fold, and maple leaf extract (at 3% v/v) also increased astaxanthin yield significantly (above 7 mg/L) compared to the control (5.8 mg/L) (Kothari et al., 2019). Various vegetable extracts, including *Perilla frutescens* (final concentration, 5%) or *Allium fistulosum* (final concentration, 1%), could significantly improve cell growth and astaxanthin yield in *P. rhodozyma* (Kim et al., 2007). Phytohormones, which are signaling molecules derived from plants, play a crucial role not only in the physiological and metabolic regulation of higher plants but also in stimulating a wide range of biological processes. These processes include cell growth and the metabolism of fatty acids, carotenoids, and carbohydrates in microalgae and fungi. 6-benzylaminopurin (6-BAP) was found to increase biomass and astaxanthin production in *P. rhodozyma* by more than 20% at a concentration of down to 0.25 mg/L. Metabolome analysis showed that 6-BAP enforced glucose uptake and glycolysis and inhibited the TCA cycle to improve astaxanthin synthesis. Moreover, 6-BAP induced gene expressions of key enzymes in the carotenogenic pathway to increase astaxanthin production (Pan et al., 2020). A high concentration of 500 mg/L gibberellic acid (GA) increased astaxanthin biosynthesis in *P. rhodozyma* by 77% in the fed-batch system. Astaxanthin synthase (*crtS*) that catalyzes the conversion of β-carotene to astaxanthin was induced while genes of the steroid biosynthetic pathway were suppressed by GA (Liu et al., 2022). Although phytohormone has been proved as an ideal strategy to improve the astaxanthin productivity in *P. rhodozyma*, the research on mechanism of phytohormone inducing astaxanthin synthesis is still at an early stage with much detail unclear.

Salicylic acid (SA) is an important phytohormone in the defense networks of high plants, macroalgae and microalgae. Effects of SA on growth and astaxanthin were mainly focused on the microalgae *Haematococcus pluvialis* (Raman and Ravi, 2011; Gao et al., 2015; Yu et al., 2015). As Raman et al. reported, SA (100 μM) could increase the astaxanthin content by 6.8-fold under a low light condition through improving the activities of superoxide dismutase and ascorbate peroxidase (APX) by 4.5- and 15.5-fold, respectively. This result indicated that oxidative stress induced by SA improved production of antioxidants such as astaxanthin in *H. pluvialis* (Raman and Ravi, 2011). Gao et al. found that SA changed the expressing patterns of 26 transcription factors (TFs), 12 of which belongs to the plant-specific TFs, to improve astaxanthin production in *H. pluvialis* (Gao et al., 2015). SA was also found to induce higher transcriptional expression levels of the carotenogenic genes, including *ipi-1*, *ipi-2*, *psy*, *pds*, *lyc*, *bkt2*, *crtR-B* and *crtO*, resulting in higher astaxanthin productivity in *H. pluvialis* (Gao et al., 2012). However, the global mechanisms of SA regulating astaxanthin synthesis in microorganisms are still unclear, and the effect and mechanism of SA on cell growth and astaxanthin production in *P. rhodozyma* have never been reported.

In this study, we analyzed the effects of SA on biomass and astaxanthin accumulation in *P. rhodozyma* and found that SA at an extremely low concentration could improve its cell growth, astaxanthin content and yield. Using the transcriptomic method, the global transcriptional profiles of the *P. rhodozyma* cells between the SA-treatment and SA-free groups are compared, and the differentially expressed genes (DEGs) between the two groups are identified and analyzed. At the cellular global level, the mechanism of SA regulating the cell growth and astaxanthin accumulation in *P. rhodozyma* are presented and illuminated in this study. A upregulated DEG, polyamine transporter gene (*PT*), was overexpressed in the *P. rhodozyma* to obtain the yeast transformant Prh-PT-006. The biomass, astaxanthin content and yield of strain Prh-PT-006 under SA-treatment condition were evaluated, and the SA-treatment combining with genetic modification strategy for astaxanthin production was constructed. Our result will deepen the theoretic system of microbial astaxanthin synthesis and regulation, provide the engineering targets for constructing astaxanthin high-yield strains, and lay solid foundation for accelerating the industrialization production of microbial astaxanthin.

## 2 Materials and methods

### 2.1 Chemicals

Astaxanthin and salicylic acid of HPLC grade were purchased from Sigma-Aldrich, Inc. (St. Louis, MO, United States). All other chemicals were sourced from Aladdin Biochemical Technology Co., Ltd. (Shanghai, China) and met at least analytical-grade standards.

### 2.2 Microbial strains and culture condition

The *P*. *rhodozyma* strain AS2.1557 was obtained from the China General Microbiological Culture Collection Center (Beijing, China). The YPD medium was composed of 20 g/L glucose, 10 g/L yeast extract and 20 g/L peptone. The YM medium was composed of 5 g/L tryptone, 3 g/L yeast extract, 3 g/L malt extract, and 10 g/L glucose. The yeast culture condition was at 22°C and 300 rpm for 96 h.

### 2.3 Salicylic acid- or G418-treatment

Salicylic acid (SA) was dissolved in the deionized water and added to the YPD medium with the final concentrations of 0.25 mg/L, 0.5 mg/L, 1 mg/L, 2 mg/L and 3 mg/L, respectively (SA-treatment). Similar amounts of deionized water were added to the YPD medium and used as the controls (SA-free). To select the positive transformants overexpressing the *PT* gene, YM medium plate containing 50 mg/L G418 was prepared on which transformants were cultured.

### 2.4 Biomass measurement

The *P. rhodozyma* AS2.1557 biomass was measured in terms of dry cell weight (DCW). An aliquot of 50 mL of the cell culture was centrifuged at 4°C and 7,000 g for 5 min and then washed twice with distilled water. The cell pellets were lyophilized to a constant weight at −50°C for approximately 48 h and weighed (Yu et al., 2019).

### 2.5 Astaxanthin extraction and analysis

A volume of 3 mL dimethyl sulfoxide (DMSO) was preheated at 60°C and then mixed well with the *P. rhodozyma* cell. The mixture was incubated at 50°C for 5 min. A volume of 3 mL anhydrous ethanol was added to the above mixture and incubated for 20 min. Then, the sample was extracted by ultrasonic for 10 min, and centrifuged at 8000 rpm and 4°C for 10 min. The extraction procedure was performed for several cycles until the cell pellet turned white. The supernatants were collected and diluted to 20 mL. The astaxanthin was analyzed on an Essentia LC-16 HPLC system equipped with a Hypersil BDS C18 column (Shimadzu, Japan). The UV detection wavelength of astaxanthin, injection volume, column temperature and flow rate were 478 nm, 10 μL, 25°C and 1 mL/min, respectively. The mobile phase was composed of methanol and acetonitrile with a volume ratio of 9:1 (Pan et al., 2020).

### 2.6 RNA extraction and library construction

The total RNA was extracted from each sample using TRIzol Reagent (Invitrogen, United States). The RNA amount and purity were analyzed by an Agilent 2100 Bioanalyzer (Agilent Technologies, United States), a NanoDrop apparatus (Thermo Fisher Scientific Inc., United States), and 1% agarose gel, respectively. The RNA integrity was assessed by Bioanalyzer 2100 (Agilent Technologies, United States) with the RIN number higher than 7.0, and confirmed by electrophoresis with denaturing agarose gel (Yu et al., 2019). Poly (A) RNA was purified using Dynabeads Oligo (dT)25-61005 (Thermo Fisher, United States) and two cycles of purification. Poly (A) RNA was used as template and the 1st strand cDNA was synthesized using ProtoScript II Reverse Transcriptase (Invitrogen, United States). The 2nd strand cDNA was synthesized using Second Strand Synthesis Enzyme Mix (including dACG-TP/dUTP), *Escherichia coli* DNA polymerase I (NEB, United States), RNase H (NEB, United States) and dUTP Solution (Thermo Fisher, United States). An A-base was added to each strand, and an adaptor with a T-tailed was ligated. The size selection of Adaptor-ligated DNA was then performed using AMPureXP beads. After the Uracil-Specific Excision Reagent (USER) enzyme (NEB, United States) treatment of the U-labeled second-stranded DNAs, the ligated products were amplified with PCR for 10 cycles with both primers carrying sequences which can anneal with the flow cell to perform bridge PCR. The PCR products were cleaned up using AxyPrep Mag PCR Clean-up (Axygen, United States), validated using an Agilent 2100 Bioanalyzer (Agilent Technologies, United States), and quantified by a Qubit 2.0 Fluorometer (Invitrogen, United States). The average insert size was 300 ± 50 bp. Thus, 2 × 150 bp paired-end sequencing (PE150) was performed on an Illumina HiSeq instrument following the vendor’s recommended protocol (Illumina, United States).

### 2.7 RNA-seq data analysis

The FASTAQ format data was first processed with the fastap software (https://github.com/OpenGene/fastp), and the clean reads with adapters, poly N, and low-quality reads removed were obtained. The Q20, Q30, GC-content were calculated, and the further analysis was based on the clean data with high quality. The HISAT2 (https://ccb.jhu.edu/software/hisat2) was used to map reads to the reference genome of.

*P. rhodozyma* CBS6938 (Genebank No. GCA_014706385.1). StringTie was used to estimate the expression levels of all transcripts through the FPKM value. The differentially expressed genes (DEGs) were selected with fold change >1.5 or fold change <0.5 and *p*-value (parametric F-test comparing nested linear models) < 0.05 by R package edgeR (https://bioconductor.org/packages/release/bioc/html/edgeR.html). The R package GOseq was used to conduct GO enrichment analysis of the DEGs with a significant *p*-value < 0.05 (Li and Dewey, 2011). The KOBAS software was used to enrich significant DEGs in the KEGG pathway (Kyoto Gene and Genomic Encyclopedia, http://en.wikipedia.org/wiki/KEGG) (Mao et al., 2005).

### 2.8 Quantitative real-time reverse transcription PCR (RT-PCR)

The expression levels of selected 33 DEGs from the transcriptomic data were further validated through qRT-PCR. The gene-specific primer were designed by Primer Premier 5.0 and were listed in Supplementary Table S1. The total RNA was extracted from samples using the method described above. qRT-PCR was performed with the CFX96 Touch qRT-PCR system (BIORAD, United States). The PCR conditions were: 98°C for 30 s, 40 cycles of 98°C for 5 s, and 58°C for 34 s. The 18S rRNA gene was used as an internal standard, and the relative quantitative method (ΔΔCt) was used to evaluate the relative quantitative variations (Livak and Schmittgen, 2001). Every qRT-PCR reaction was performed in triplicate, and the data were normalized using the average for the internal standard. The linear correlations of the DGEs expression levels deduced from the transcriptomic data and RT-PCR were fitted with *R* ^2^ value to validate the transcriptomic results.

### 2.9 Construction of the polyamine transporter gene (*PT*)-overexpressing vector

An overlap PCR technology was used to construct a polyamine transporter gene (*PT*)-overexpressing vector with minor modifications (Yu et al., 2020). The vector with a 7097bp-length contains 18sup, Pgdp, G418, Tgdp, Padh4, PT, Tact and 18sdown in sequence, represented as upstream of the 18SrDNA, gdp promoter, G418-resistence gene, gdp terminator, adh4 promoter, polyamine transporter gene, act terminator and downstream of the 18SrDNA, respectively. This vector is integrated into the 18SrDNA location of *P. rhodozyma* genome through double-cross homologous recombination with resistance to G418 as selective marker and overexpression of PT gene under the strong promoter Padh4 (Hara et al., 2014a). The 8 DNA fragments were firstly amplified separately, with the genome of *P. rhodozyma* as template for 18sup, 18sdown, Pgdp, Tgdp, Padh4, PT, Tact, and the pPIC9K plasmid as template for G418. The primers were designed by the Primer Premier Ver. 6.0 (PREMIER Biosoft., United States), and are listed in Supplementary Table S1. The same oligonucleotides with upstream and downstream fragments were added into the F- and R-primers to make the 8 fragments ligated in sequence by the second step overlap PCR. In the second-step PCR, the first-step 8 PCR products, were as the template with an approximate mol ratio of 18sup, Pgdp, G418, Tgdp, Padh4, PT, Tact, 18sdown as 1:3:5:7:7:5:3:1, respectively, and 18sup-F and 18sdown-R as the primers. The PCR condition was a cycle of 98°C for 3 min, followed by 30 cycles (98°C for 10 s, 55°C for 1 min and 72°C for 3 min), and a final cycle of 10 min at 72°C.

### 2.10 Genetic transformation of the PT-overexpressing vector into *P. rhodozyma* cells

The transformation of *P. rhodozyma* cells were based on the electro-transformation method reported by Niklitschek et al. with minor modifications (Niklitschek et al., 2008). The yeast was cultured on YM medium (OD_660nm_ = 1.5) and cells were harvested at 10,000 g for 5 min. The pellet was suspended in the potassium phosphate buffer (50 mM potassium phosphate, pH 7, 25 mM DTT) and incubated at 21 °C for 15 min. Then, the cells were washed twice with the STM buffer (270 mM sucrose, 10 mM Tris HCl, 1 mM MgCl, pH 7.5) and suspended with 500 μL STM buffer at 4 °C as competent cell. A volume of 60 μL competent cell and 10 μg DNA fragment were mixed well and the electroporation conditions was 25 μF, 1,000 Ω, 800 V using a Gene Pulser (BioRad, United States). The transformants were selected on YM with 50 mg/L G418.

### 2.11 Confirmation of *PT*’s integration into *P. rhodozyma* genome and its expression

The *PT* gene’s integration was further confirmed in the transformants with G418 resistance through PCR technology, and the *PT* gene’s expression was confirmed by RT-PCR technology. Genomes of *P. rhodozyma* wild strain and transformants were extracted using TaKaRa MiniBEST universal Genomic DNA Extraction Kit based on manufacturer’s instruction (Takara Bio, Dalian, China). PCR was performed using the extracted genomes as templates and Confirm-F and Confirm-R as primers (Supplementary Table S1). The PCR condition was described above. The RT-PCR was described as the 2.8 section.

### 2.12 Statistical analysis

All experiments were repeated three times, and data were expressed as mean values ± standard deviations (SD). Data were statistically analyzed using one-way ANOVA and Scheffe’s test using SPSS^®^ 20.0 (SPSS Inc., Chicago, IL, United States).

## 3 Results

### 3.1 Trace salicylic acid stimulates cell growth and astaxanthin biosynthesis

**FIGURE 1:**
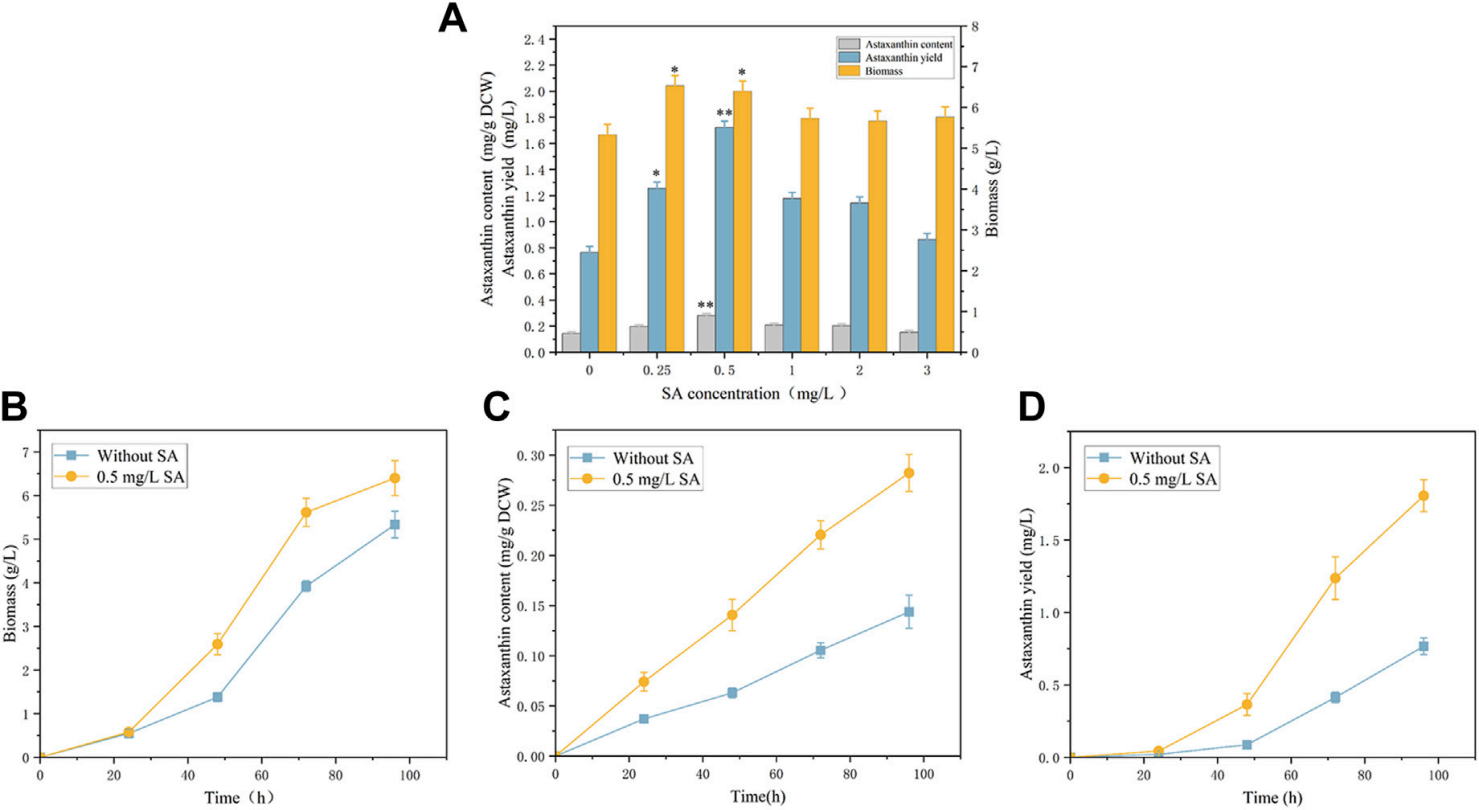
Effects of salicylic acid (SA) on biomass and astaxanthin accumulation in *Phaffia rhodozyma*. **(A)** Effects of different concentrations of SA (0 mg/L, 0.25 mg/L, 0.5 mg/L, 1 mg/L, 2 mg/L and 3 mg/L) on biomass, astaxanthin content and yield in *P. rhodozyma*. **(B)** 96 h time-course effect of 0.5 mg/L SA on biomass in *P. rhodozyma*. **(C)** 96 h time-course effect of 0.5 mg/L SA on astaxanthin content in *P. rhodozyma*. **(D)** 96 h time-course effect of 0.5 mg/L SA on astaxanthin yield in *P. rhodozyma*. Data are given as means ± SD, *n* = 3. Asterisk without the indicator line compared with the WT *p < 0.05 **p < 0.01.

We analyzed effects of exogenous phytohormone salicylic acid (SA) on biomass and astaxanthin production (Figure 1). It is worth noting that the *P. rhodozyma* cell was very sensitive to SA, and an extremely low concentration range of 0.25–3 mg/L SA could improve this yeast’s biomass, astaxanthin content and yield by 6.3%–22.5%, 6.6%–96.1% and 12.9%–125.4%, respectively. Under the optimal SA treatment condition (0.5 mg/L), the *P. rhodozyma* biomass, astaxanthin content and yield reached 6.4 g/L, 0.28 mg/g DCW and 1.7 mg/L, with 20%, 96.1% and 125.4% higher than those from the control group (the SA-free group) (Figure 1A). The time-course effects of 0.5 mg/L SA on biomass, astaxanthin content and yield, were also analyzed (Figures 1B–D). The results showed that the biomass, astaxanthin content and yield from the SA treatment group at 24 h, 48 h, 72 h and 96 h, except the biomass at 24 h, were significantly distinguished and improved comparing with those from the control group (SA-free group). These results indicated that *P. rhodozyma* cell response the SA treatment to improve its biomass, astaxanthin content and yield. The optimal 0.5 mg/L of SA was selected to further analyze the transcriptomic mechanism of SA inducing cell growth, astaxanthin content and yield in *P. rhodozyma*.

### 3.2 Illumina sequencing, reads mapping, and statistical analysis

The total RNA was extracted from *P. rhodozyma* cells with and without 0.5 mg/L SA treatment, representing the S and C group, respectively. The RNA-seq libraries were constructed and sequenced. The raw data was processed to a range of approximate 35.2–49.9 million valid reads (5.28G-7.49G) with high Q20 (Sequencing error rate <0.01) and Q30 (Sequencing error rate <0.001) values for each sample (Supplementary Table S2). These results indicated that the sequencing data has high quality and credibility and can be further analyzed. The reference genome sequence of *P. rhodozyma* has been released (NCBI number GCA_001007165.2_Xden1), thus, the valid reads were mapped into the reference genome. Based on the gtf file of reference genome for annotation, the sequencing data mapped to the genome and their distribution in genome region were analyzed (Supplementary Table S3). In all groups, a range of 95.85%–97.58% valid reads could be mapped in the reference genome with 93.96%–95.65% of unique mapped reads and 1.84%–1.94% of multi mapped reads. These results showed that most of our sequencing data could be mapped to the reference genome, and our data had high quality and credibility and could be further analyzed.

### 3.3 Changes in transcriptional profile induced by SA treatment in *P. rhodozyma*

**FIGURE 2:**
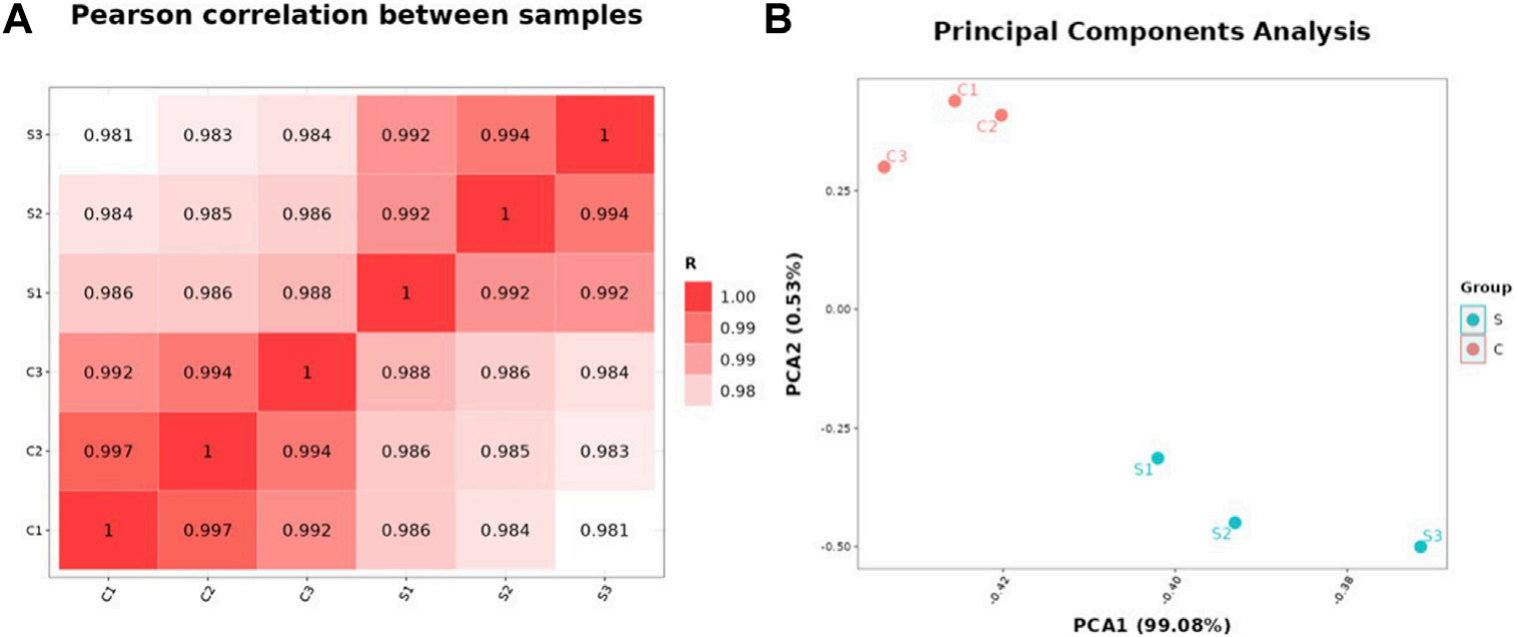
Transcriptomic profiles of the *P. rhodozyma* cells under the SA-treatment (S group) and SA-free (C group) conditions. **(A)** Pearson correlation between the samples from the S and C groups (The closer the R-value is to 1, the more similar transcriptomic profiles of the two samples are). **(B)** PCA analysis of the samples from the S and C groups (The closer the points representing the samples are, the more similar the transcriptomic profiles between the samples are).

**FIGURE 3:**
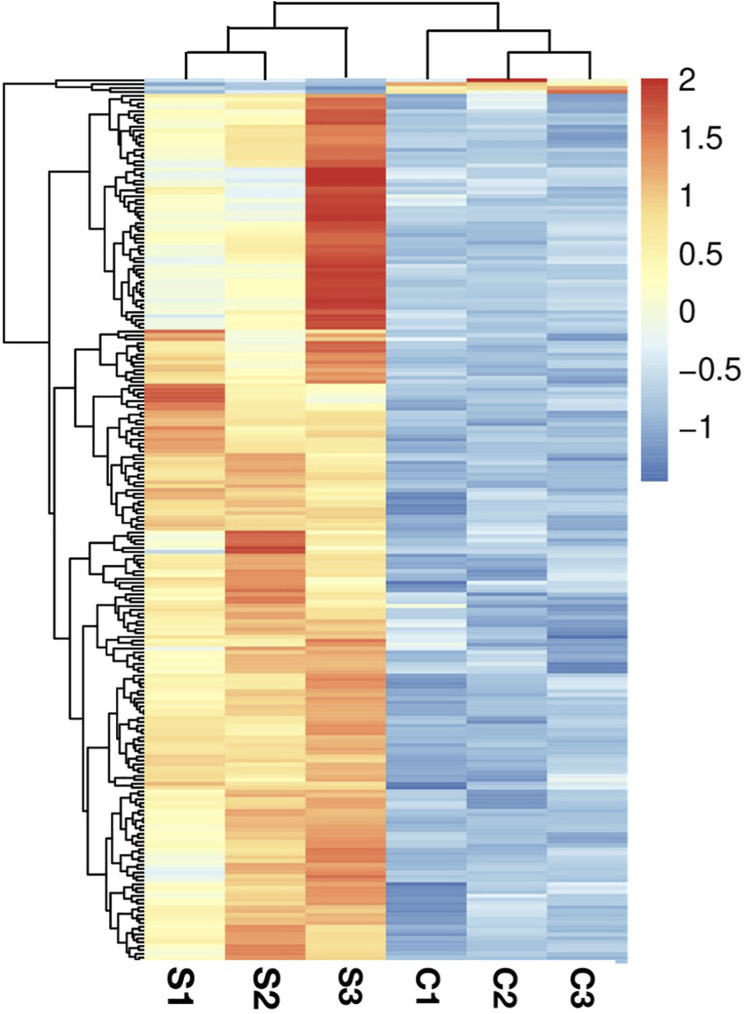
Hierarchical Cluster Analysis (HCA) analysis based on all transcripts from the S and C groups.

Pearson correlation and principal components analysis (PCA) were performed to analyze the transcriptomic profile between the SA-treatment and SA-free groups (the S and C group). The pearson correlation analysis showed that the S- and C- group samples had lower correlation while the samples in the same group had higher correlation (Figure 2A). The PCA results showed that the profiles of all gene transcripts with and without SA treatments (the S and C groups) can be well discriminated at the PCA1 level, which could explain the 99.08% difference (Figure 2B). A further Hierarchical Cluster Analysis (HCA) based on all gene transcripts from the S and C groups showed that the transcriptomic profiles of the two groups samples were obviously branched (Figure 3). These results suggested that SA indeed significantly disturbed the expressions of genes at the global cellular level in *P. rhodozyma*, which corresponded to the result that SA could induce the cell growth and astaxanthin biosynthesis (Figure 1). Thus, the mechanism of SA disturbing the transcriptomic profiles of *P. rhodozyma* should be further analyzed to uncover mechanism of SA inducing the cell growth and astaxanthin biosynthesis.

### 3.4 DEGs identification, GO function annotation and KEGG pathway enrichment

**FIGURE 4:**
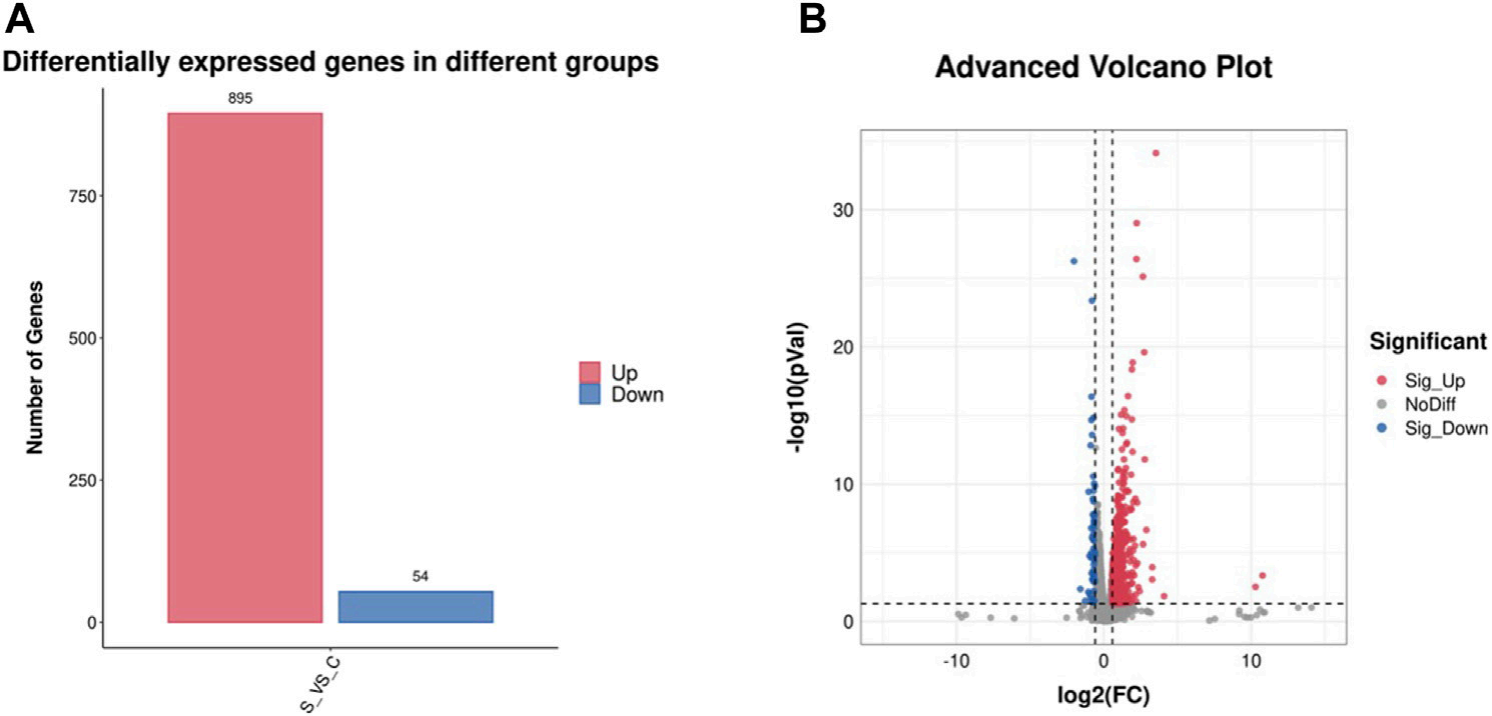
The differentially expressed genes (DEGs) number **(A)** and volcano plot **(B)**.

**FIGURE 5:**
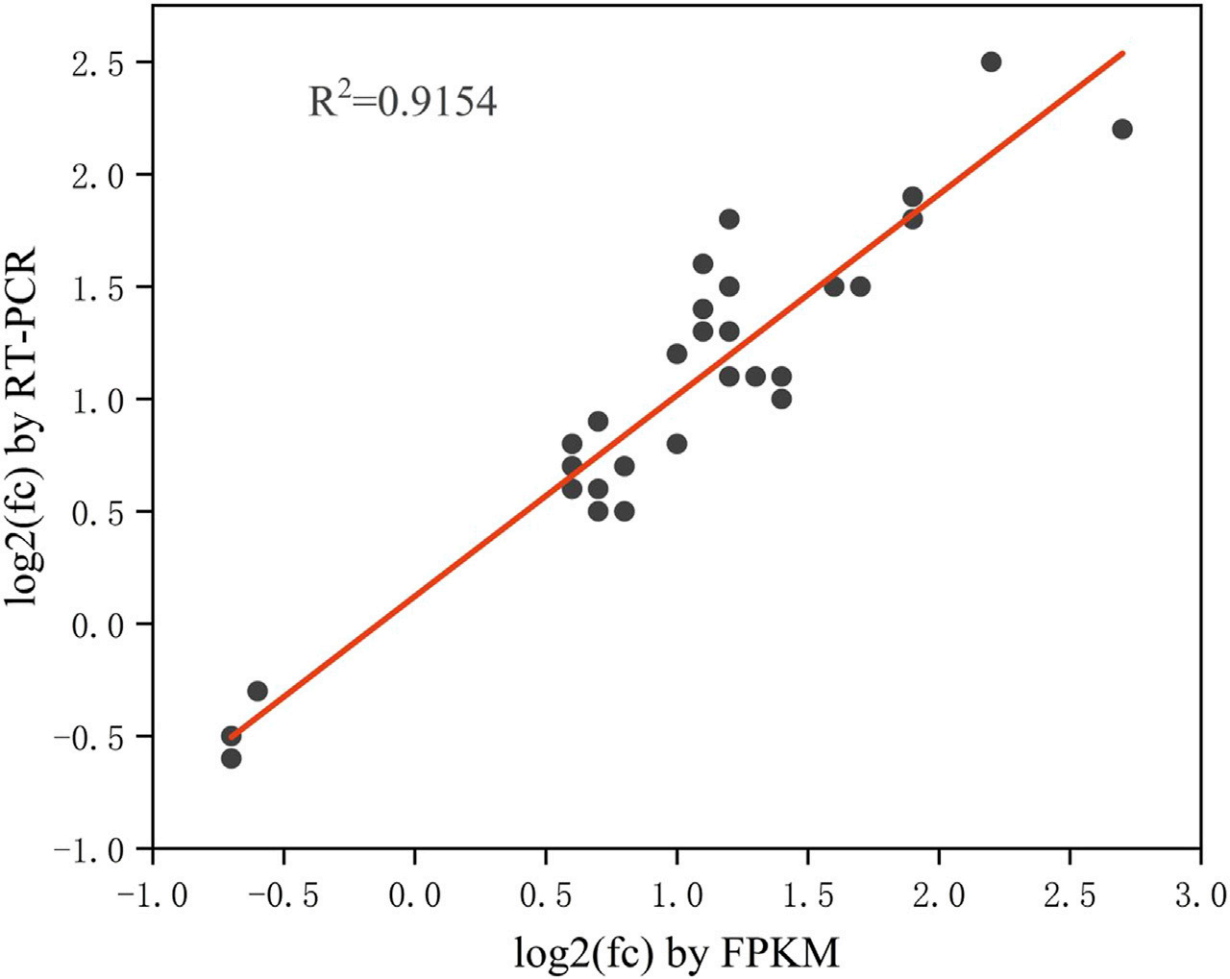
The linear relationship between the 33 DEGs results from RT-PCR and FPKM (The closer the R-value is to 1, the stronger the linear relationship between the two sets of data).

The differentially expressed genes (DEGs) with fold change >1.5 or fold change <0.5 and parametric F-test comparing nested linear models (*p*-value <0.05) were identified to uncover the mechanism of SA inducing cell growth and astaxanthin biosynthesis. A total of 949 DEGs with 895 upregulated and 54 downregulated was identified in *P. rhodozyma* cells of the SA-treatment group (S group) compared to those SA-free group (C group) (Figure 4). To validate the results from transcriptomic data, a total of 33 up- or downregulated DEGs were selected, and their expression levels were analyzed using RT-PCR technology (Figure 5; Table 1). The linear correlation of the results from FPKM and RT-PCR was fitted with a *R* ^2^ value up to 0.9154, indicating that the results derived from the two methods had a higher linear relationship and our transcriptomic data had higher accuracy and credibility (Figure 5).

**TABLE 1:** The DEGs involved in regulation of SA on biomass and astaxanthin synthesis in *P*. *rhodozyma*.

Group	Gene ID	Gene description	FPKM			log2(fc) by RT-PCR
			SA treatment	Control	log2(fc)	
Astacanthin metabolism	2447	astaxanthin synthase	88.2 ± 9.5^a^	56.3 ± 14.2	1↑	1.2↑
Central carbon metabolism	11,901	pyruvate kinase	104.3 ± 15.3^b^	41.9 ± 2.9	1.3↑	1.1↑
	8393	isocitrate dehydrogenase	185.5^b^	82.5	1.2↑	1.1↑
Fatty acid metabolism	10,215	cytochrome P450	0.8 ± 0.1^b^	0.3 ± 0.1	1.2↑	1.3↑
	10,399	fatty acid synthase	131.8 ± 28.4^a^	197.7 ± 19.5	0.7↓	0.5↓
Sterol metabolism	2377	hydroxymethylglutaryl-CoA synthase	466.5 ± 39.5^a^	771.2 ± 90.4	0.7↓	0.6↓
Anti-stress metabolism	9309	glutathione S-transferase	187.8 ± 17.3^b^	40.4 ± 9.5	2.2↑	2.5↑
	11,321	catalase	48.9 ± 6.8^a^	31.5 ± 9.3	0.6↑	0.8↑
	7839	peroxiredoxin	3.1 ± 0.6^a^	1.2 ± 0.4	0.6↑	0.6↑
	3693	thioredoxin	30.8 ± 9.4^b^	14.6 ± 2.7	1.1↑	1.6↑
	7785	spermine/spermidine synthase	13.6 ± 1.6^b^	5.9 ± 0.6	1.2↑	1.5↑
Transportation	4787	copper transporter	55.1 ± 9.3^b^	14.7 ± 2.6	1.9↑	1.9↑
	3671	amino acid transporters	65.7 ± 19.2^b^	22.3 ± 2.6	1.7↑	1.5↑
	11,455	amino acid permease	19 ± 2.6^b^	5.1 ± 0.6	1.9↑	1.8↑
	11,091	uridine permease/thiamine transporter	162.1 ± 25.1^b^	63.5 ± 8.4	1.4↑	1.1↑
	625	abc transporter	177.2 ± 18.2^b^	82.6 ± 12.3	1.1↑	1.3↑
	11,031	oligopeptide transporter	12.1 ± 0.4^a^	19.6 ± 2.5	0.7↓	0.5↓
	4151	polyamine transporter	63.3 ± 14.2^b^	10 ± 3.6	2.7↑	2.2↑
	12,365	magnesium transporter	25.7 ± 2.6^a^	15.6 ± 0.6	0.7↑	0.5↑
	7263	gamma-aminobutyrate permease	48.5 ± 3.6^a^	30.4 ± 4.7	0.7↑	0.9↑
	8291	s-adenosylmethionine transporter	36.8 ± 5.2^b^	17.2 ± 2.4	1.1↑	1.4↑
	5465	Fluoride ion transporter	9.9 ± 0.4^a^	5.9 ± 1.2	0.8↑	0.5↑
DNA metabolism	12,339	thymidylate synthase	73.9 ± 29.1^a^	111.9 ± 17.3	0.6↓	0.3↓
	1329	inosine triphosphate pyrophosphatase	56.6 ± 9.5^a^	36.1 ± 4.6	0.6↑	0.7↑
	8807	thymidylate kinase	7.4 ± 1.3^a^	4.4 ± 2.3	0.7↑	0.6↑
	11,695	guanylate kinase	6.7 ± 0.6^b^	2.3 ± 0.3	1.6↑	1.5↑
	8869	glutamine synthetase	12.3 ± 1.5^a^	6.9 ± 0.6	0.8↑	0.7↑
Signal transduction	6847	Ca/calmodulin-dependent protein kinase	3.2 ± 0.9^b^	1.4 ± 0.4	1.2↑	1.8↑
	9597	srf-type TF	26.9 ± 3.6^a^	17.1 ± 4.8	0.7↑	0.6↑
	10,943	ste3	0.8 ± 0.1^b^	0.3 ± 0.1	1.4↑	1.0↑
	3687	sho1	9.4 ± 2.5^a^	6.0 ± 3.6	0.7↑	0.5↑
	11,761	basic-leucine zipper TF	810.9 ± 35.2^a^	1349.3 ± 90.4	0.7↓	0.5↓
	9587	basic helix-loop-helix (bHLH) TF	101.4 ± 21.4^a^	50.8 ± 17.8	1↑	0.8↑

Data are given as means ± SD, n = 3.

^a^ *p* < 0.05 compared with the control.

^b^ *p* < 0.01 compared with the control.

**FIGURE 6:**
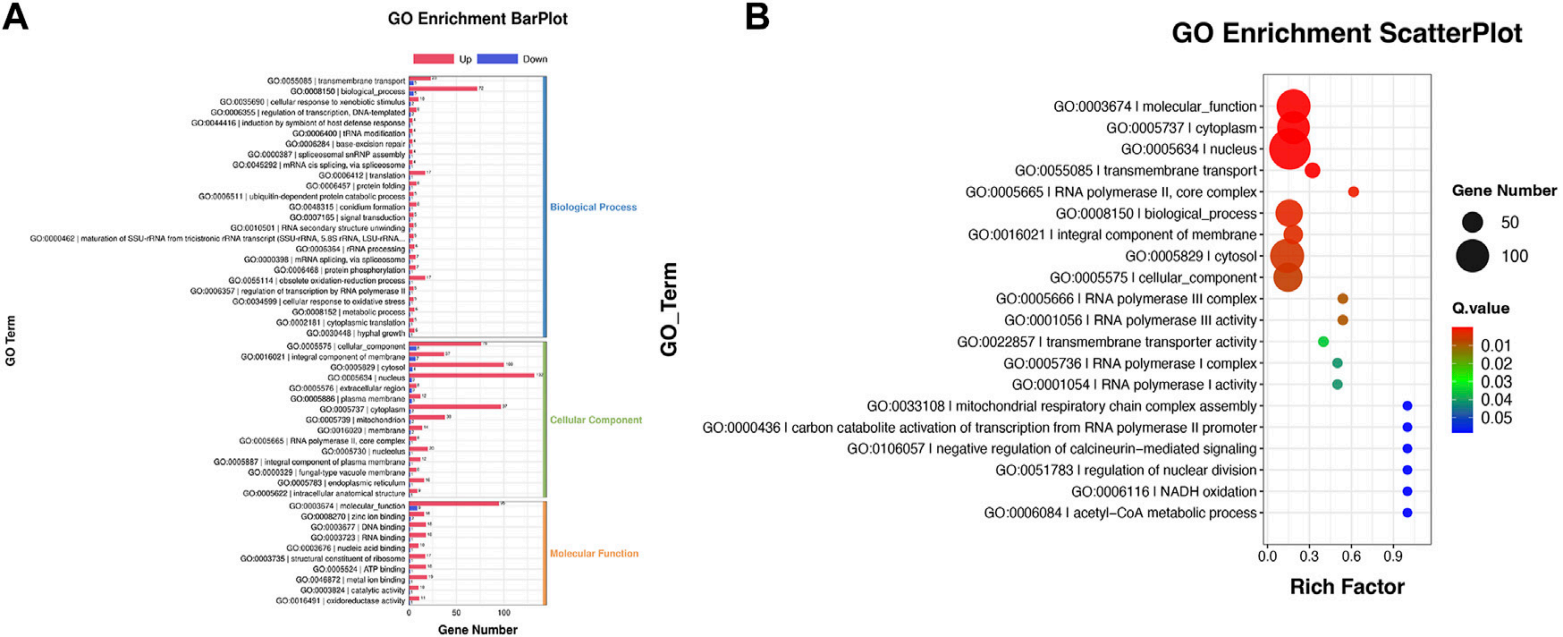
GO annotation of the DEGs between the cells from SA-treatment and SA-free conditions. **(A)** GO enrichment BarPlot. The numbers on the bar chart represent the number of DEGs in every GO term. **(B)** GO enrichment ScatterPlot. The circle sizes represent DEGs number in every GO term, the various colors represent the rich significance.

**FIGURE 7:**
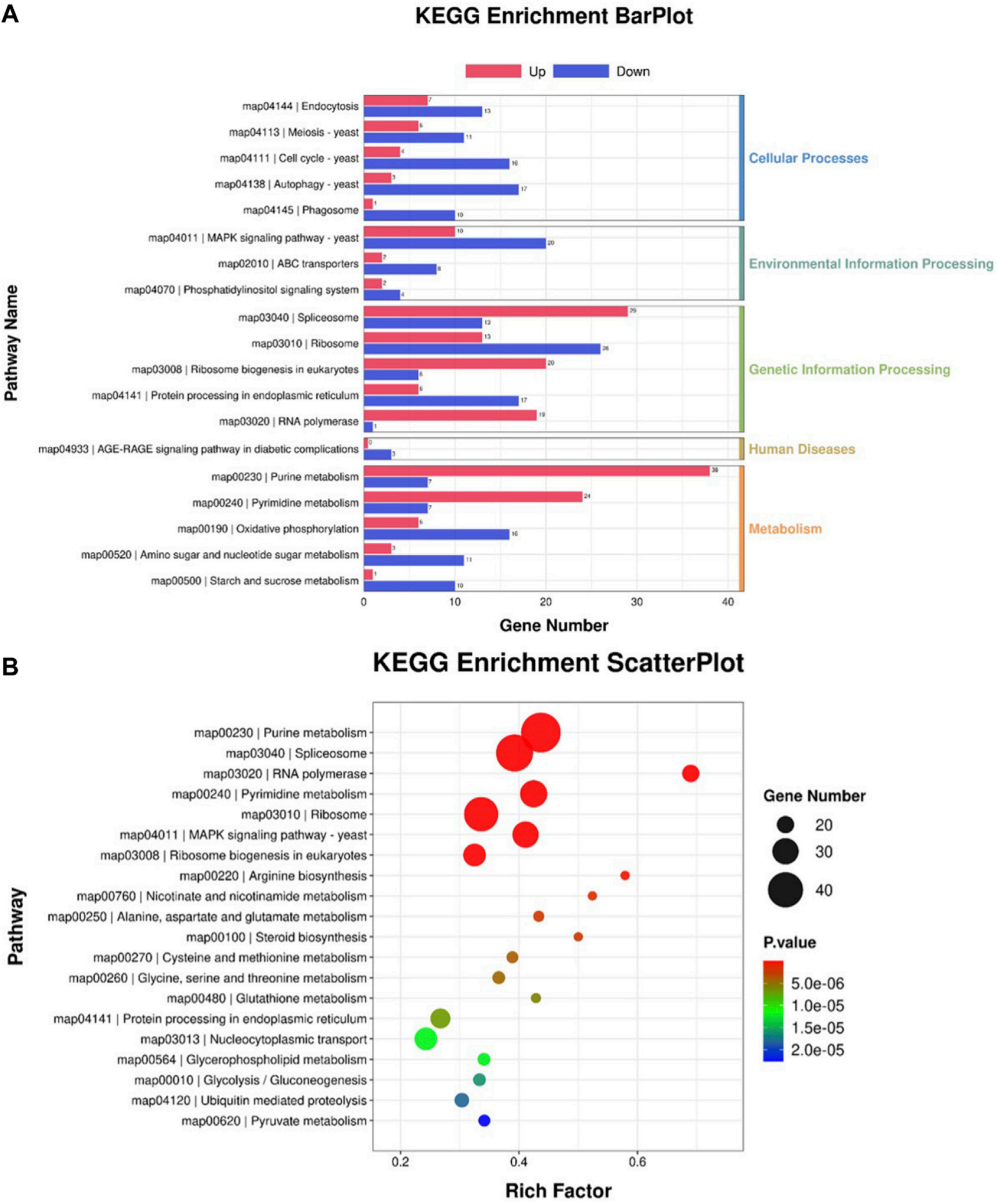
KEGG annotation of the DEGs between the cells from SA-treatment and SA-free conditions. **(A)** KEGG enrichment BarPlot. The numbers on the bar chart represent the number of DEGs in every KEGG term. **(B)** KEGG enrichment ScatterPlot. The circle sizes represent DEGs number in every KEGG term, the various colors represent the rich significance.

Reannotated to GO terms, the DEGs were divided into 50 categories under three GO domains of molecular function (MF), cellular component (CC), and biological process (BP), respectively (Figure 6A). A total of 735 DEGs were annotated as various GO terms, including “molecular function”, “cytoplasm”, “nucleus”, “transmembrane transport”, “RNA polymerase”, “biological process”, “integral component of membrane”, “cytosol”, “cellular component”, “transmembrane transporter activity”, “acetyl-CoA metabolic process” and “NADH oxidation” with *p*-value < 0.001 (Figure 6B). Meanwhile, DEGs were enriched into 19 functional KEGG groups of the “cellular processes”, “environmental information processing”, “genetic information processing”, “human disease” and “metabolism” (Figure 7A). A total of 788 DEGs were categorized into the pathways, including “purine metabolism”, “pyrimidine metabolism”, “spliceosome”, “nucleocytoplasmic transport”, “MAPKs signaling pathway-yeast”, “amino acid metabolism”, “steroid biosynthesis”, “Nucleocytoplasmic transport”, “Glycolysis/Gluconeogenesis”, “Pyruvate metabolism”, “ABC transporter”, “terpenoid backbone biosynthesis” and “fatty acid degradation” with *p*-value < 0.001 (Figure 7B).

### 3.5 Global regulation network induced by SA for astaxanthin accumulation

#### 3.5.1 The DEGs related to astaxanthin biosynthesis

Based on the enrichment of the DEGs to GO or KEGG database, global regulation network induced by SA for astaxanthin accumulation was further analyzed. It was found that not only astaxanthin biosynthesis, but also central carbon metabolism, fatty acid metabolism, sterol metabolism, anti-stress, transportation, DNA metabolism and signal transduction were regulated by SA treatment (Table 1). Notably, only the astaxanthin synthase (*CrtS*) in astaxanthin biosynthesis, which catalyze β-carotene to astaxanthin through an oxidation reaction, was induced by 1.5-fold under SA treatment, with the other genes related to astaxanthin biosynthesis not significantly changed (Table 1). The result suggested that SA regulated not only the astaxanthin biosynthesis pathway but also the global metabolic network to induce the cell growth and astaxanthin accumulation in *P. rhodozyma*.

#### 3.5.2 The DEGs related to the global regulation network induced by SA

In the central carbon metabolism, the genes encoding pyruvate kinase and isocitrate dehydrogenase, which catalyzes the conversion of phosphoenolpyruvate and ADP to pyruvate and ATP in glycolysis, and catalyzes isocitrate to alpha-ketoglutarate and NADPH, were upregulated to 1.3- and 1.2- fold in the SA-treatment condition, respectively (Table 1). In fatty acid metabolism, the fatty acid synthase and cytochrome P450 played important roles in fatty acid synthesis and degradation and were down- and upregulated by 0.7- and 1.2- fold under the SA treatment condition, respectively (Table 1). Sterol synthesis shares the same precursor Acetyl-CoA with astaxanthin synthesis, thus, is competitive pathway of the astaxanthin synthesis. The expression of Hydroxymethylglutaryl-CoA synthase gene, which is essential in sterol synthesis pathway, was reduced by 0.7-fold under the SA treatment condition (Table 1). SA treatment induced the glutathione S-transferase, catalase, peroxiredoxin, thioredoxin, and spermine/spermidine synthase by 2.2-, 0.6-, 0.6-, 1.1- and 1.2- fold compared to the SA-free group. The main functions of these genes are detoxication and antioxidation, thus, they were listed as the anti-stress group (Table 1). Meanwhile, SA induced the global biomolecules transportations or permeases in *P. rhodozyma*. The transporters or permeases of various biomolecules, such as metal ions, amino acid, thiamine, polyamine, gamma-aminobutyrate, S-adenosylmethionine received different degrees of induction from SA with the only exception that oligopeptide transporter was inhibited by SA. In addition, the thymidylate synthase, inosine triphosphate pyrophosphatase, thymidylate kinase, guanylate kinase and glutamine synthase, which were involved in nucleotide synthesis and divided into the DNA metabolism group, were induced by 1.2-, 0.9-, 1.6-, 0.5- and 1.7-fold by SA treatment (Table 1). Finally, SA induced the signal transductions in *P. rhodozyma*, mainly the Ca/calmodulin-dependent protein kinase, STE3, SHO1 and srf-type transcription factors (TFs) in the mitogen-activated protein kinases (MAPKs) signal pathway and the bHLH-type TF at various degrees with the basic-leucine zipper TF inhibited by 1.5-fold compared to the SA-free group (Table 1).

### 3.6 Overexpression of polyamine transporter gene further increases astaxanthin synthesis

**FIGURE 8:**
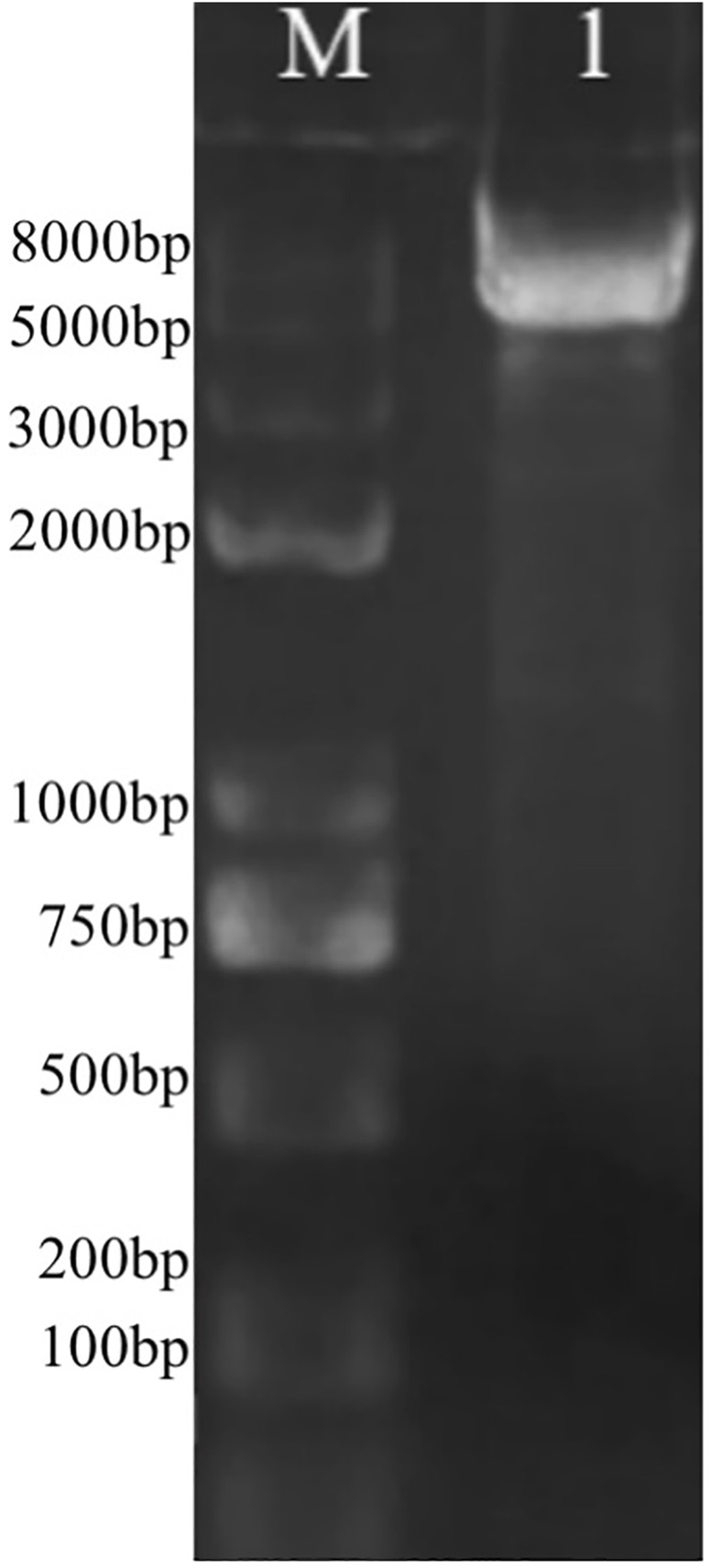
The PT-overexpressing vector through 2-step overlap PCR. M, marker; 1, the vector with a length of 7097bp containing 18sup, Pgdp, G418, Tgdp, Padh4, PT, Tact, 18sdown fragments represented as upstream of the 18SrDNA, gdp promoter, G418-resistence gene, gdp terminator, adh4 promoter, polyamine transporter gene, act terminator and downstream of the 18SrDNA, respectively.

**FIGURE 9:**
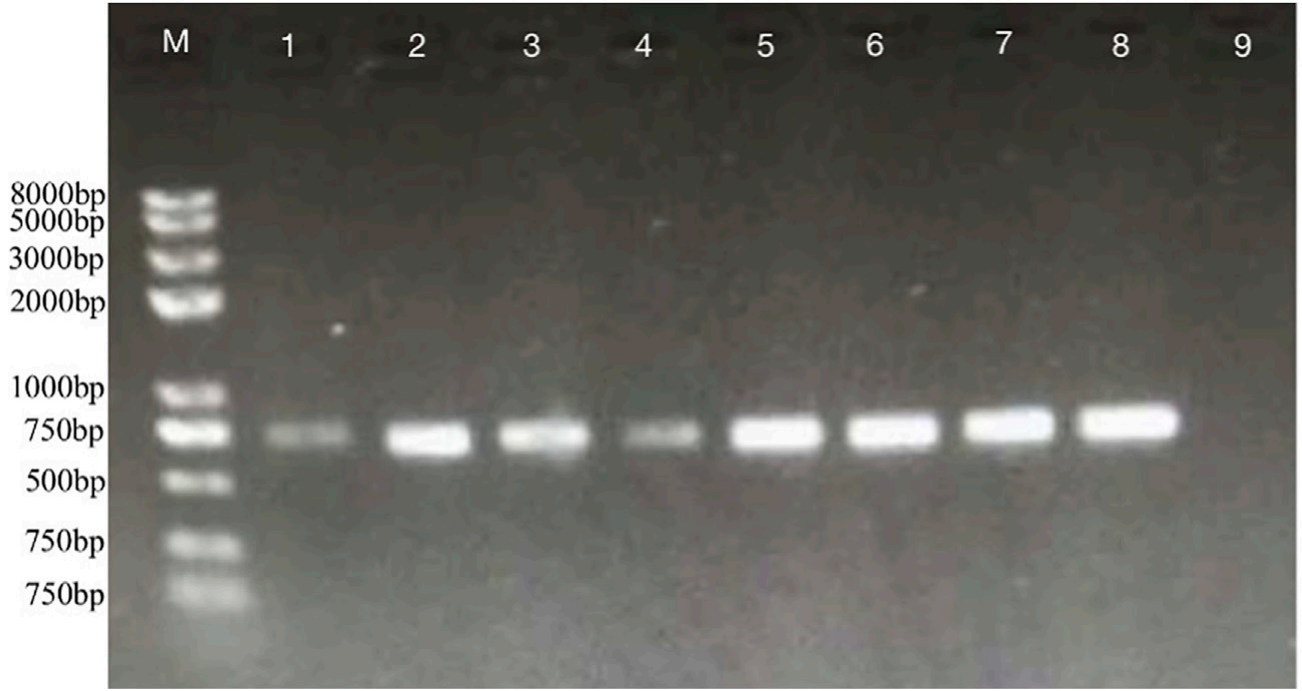
Confirmations of PT’s integrations into the 18SrDNA location of *P*. *rhodozyma* genome by PCR M, marker; lane one to eight**,** *P. rhodozyma* transformants Prh-PT-001 to Prh-PT-008 carrying the PT-overexpression vector; lane 9, the *P. rhodozyma* wild strain.

**FIGURE 10:**
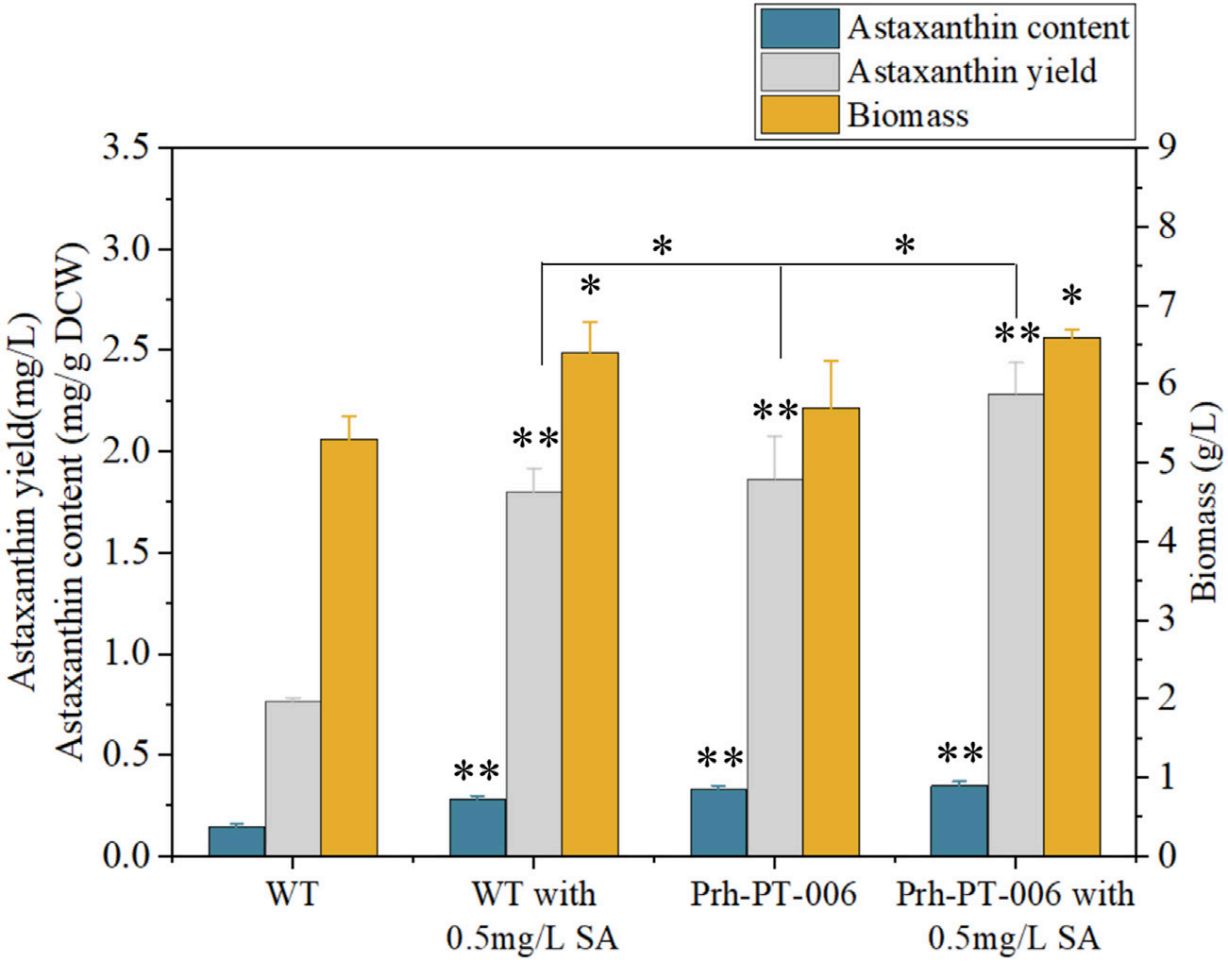
Biomass, astaxanthin content and yield of *P*. *rhodozyma* transformant Prh-PT-006 overexpressing a polyamine transporter gene under SA-free and SA-treatment conditions Data are given as means ± SD, n = 3. Asterisk without the indicator line compared with the WT *p < 0.05 **p < 0.01.

**FIGURE 11:**
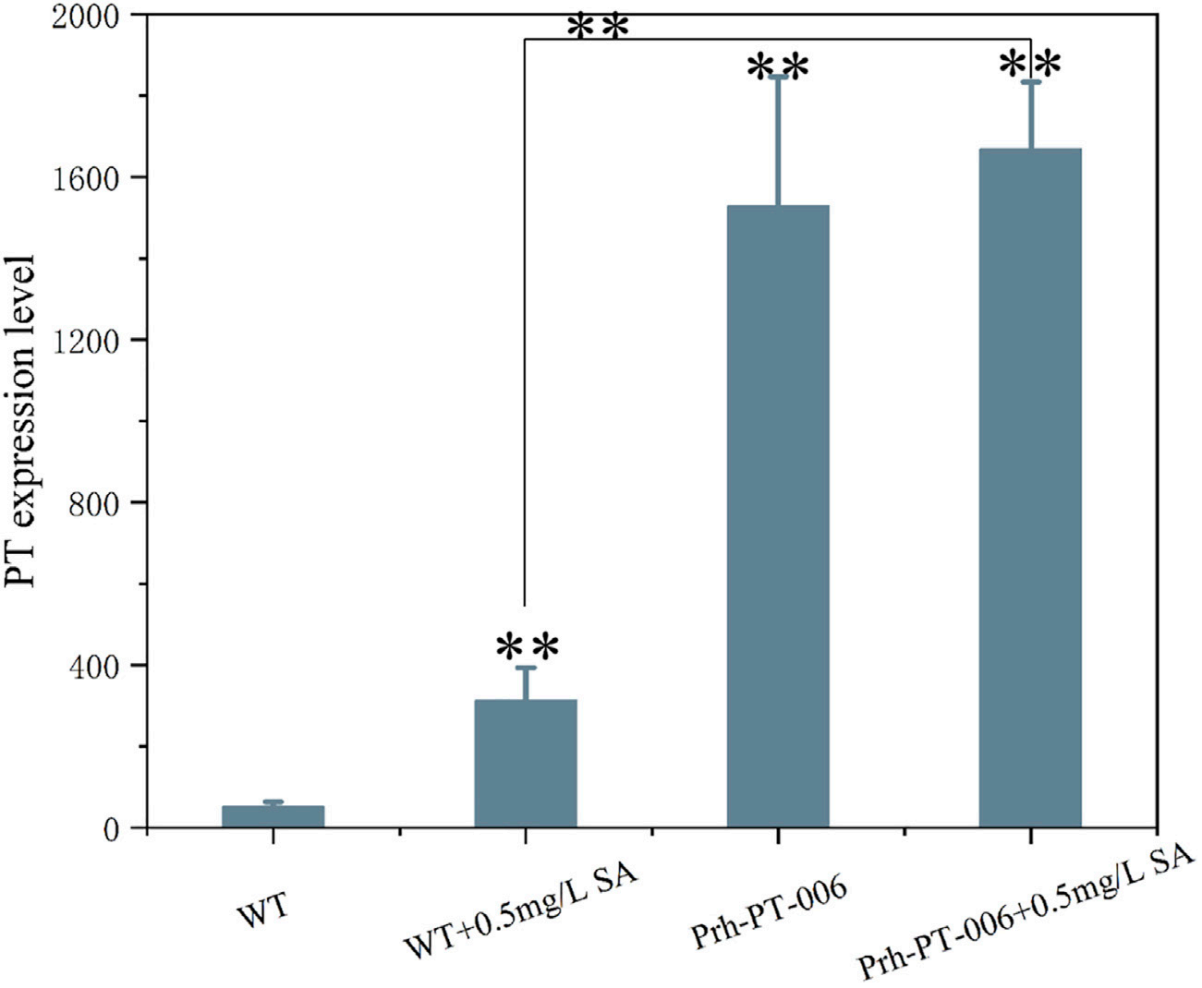
The PT gene expressions of *P*. *rhodozyma* WT and transformant Prh-PT-006 strains at the SA-free and SA-treatment condition analyzed by RT-PCR Data are given as means ± SD, n = 3. Asterisk without the indicator line compared with the WT *p < 0.05 **p < 0.01.

The DEGs induced by SA-treatment identified above are potential targets of genetic modification in *P. rhodozyma* for further astaxanthin yield improvement. Thus, we selected a polyamine transporter gene (*PT*), as a DEG with up to 2.2-fold expression induced by SA treatment, to analyze the effects of *PT* overexpression combined with SA-treatment on astaxanthin synthesis in *P. rhodozyma*. Through a 2-step overlap PCR strategy, a total of 8 DNA fragments were ligated in sequence and the PT-overexpressing vector of 7097 bp, containing 18sup, Pgdp, G418, Tgdp, Padh4, PT, Tact, 18sdown fragments with the gdp and adh4 promoters driving G418 and PT gene expressions, respectively, was obtained (Figure 8). A total of 8 transformants, Prh-PT-001 to Prh-PT-008, were randomly selected, and amplifications of a 720 bp DNA fragment covering PT, Tact and 18sdown were performed, confirming integration of the PT-overexpressing vector into the 18SrDNA location of genome (Figure 9). The transformant Prh-PT-006 was selected with the highest astaxanthin yield, and the effects of SA-treatment on biomass, astaxanthin content and yield in this transformant were analyzed. As shown in Figure 10, the biomass of transformant Prh-PT-006 under the SA-free condition was 5.7 g/L, almost identical to that of the wild strain under the SA-free condition (5.3 g/L). Meanwhile, under the SA-treatment condition, the biomass of transformant Prh-PT-006 (6.6 g/L) was almost identical to that of wild strain (6.4 g/L), indicating that overexpression of PT gene has almost no effect on cell growth of *P. rhodozyma*. However, even under the SA-free condition, the astaxanthin content in strain Prh-PT-006 were 17.0% higher than that of WT under the SA-treatment condition, confirming that the PT gene is beneficial for astaxanthin accumulation, and stronger stimulator for astaxanthin production than SA. Meanwhile, we also analyzed the synergistic effects of SA-treatment and PT gene overexpression on biomass, astaxanthin content and yield (Figure 8), and the result showed that an increase by 15.8% in biomass of transformant Prh-PT-006 induced by SA treatment with astaxanthin content almost unchanged resulted in a further increase by 22.5% in astaxanthin yield of transformant Prh-PT-006 (Figure 8). Under the SA-treatment condition, the biomass, astaxanthin content and yield of transformant Prh-PT-006 could reach 6.6 g/L, 0.35 mg/g DCW and 2.3 mg/L, 24.5%, 143.1% and 199.0% higher than the WT at the SA-free condition, respectively. In addition, the RT-PCR analysis showed that expression levels of PT gene in the Prh-PT-006 strain under the SA-free condition were up to 25.6- and 4.0- fold of those in WT under the SA-free and SA-treatment conditions, respectively (Figure 11). However, SA-treatment could not significantly increase the expression level of PT and astaxanthin content in transformant Prh-PT-006 (Figure 10; Figure 11). The explanation maybe that the promoter adh4 used for overexpression is strong enough to benefit astaxanthin synthesis, but not inducible by SA. From the other perspective, it is indicated that the PT gene is beneficial for astaxanthin production, and its expression level is positively related to astaxanthin synthesis.

## 4 Discussion

Phytohormones are kinds of signaling molecules derived from higher plants and regulate a broad spectrum of physiological processes, such as cell growth and development, biomolecule metabolism and response to environmental stresses. Phytohormones can improve crop productivity and traits, thus, they have been widely applied in the agricultural production field. Some microorganisms are closely related to higher plants, providing feasibility for a wider application of phytohormone in microbial production. For example, microalgae are microorganisms with great commercial values for its ability to produce various products, such as biofuel, nutrients, pharmaceuticals, and therapeutic compounds, and biofertilizers. Specific phytohormones can not only regulate the cell cycle and other metabolic processes in microalgae, influencing biomass and primary metabolite accumulation, but also improve the resilience of microalgae, allowing them to adapt to the prevailing conditions (Stirk and van Staden, 2020). Until now, many phytohormones, such as fulvic acid, 2, 4-epibrassinolide (EBR), strigolactone, salicylic acid (SA) and jasmonic acid (JA) have been reported to improve the biomass and astaxanthin accumulation in *H*. *pluvialis* (Gao et al., 2013; Zhao et al., 2019a; Mahadi et al., 2022). As a docosahexaenoic acid (DHA) industrial producer, *Aurantiochytrium* sp. has been reported to be induced by different phytohormones, such as 6-benzylaminopurine (6-BAP) and gibberellin, for its growth and DHA productivity (Yu et al., 2016a; Yu et al., 2016b; Yu et al., 2019; Xu et al., 2020). Like *Aurantiochytrium* sp., *P. rhodozyma* was first isolated from the plant materials which suggest it had evolved to adapt to plant metabolites, thus, phytohormones could be a great stimulator of astaxanthin production in *P. rhodozyma* (Nutakor et al., 2022). However, fewer research in *P. rhodozyma* has been reported. The effects of only gibberellic acid (GA) and 6-BAP on biomass and astaxanthin accumulation in *P. rhodozyma* have been analyzed (Pan et al., 2020; Liu et al., 2022). In this study, we found that 0.5 mg/L SA could enhance biomass, astaxanthin content and yield by 20.8%, 95.8% and 135.3%, with much less dose of SA than that in *H*. *pluvialis* (Gao et al., 2015). This result suggests that *P. rhodozyma* is more sensitive to SA than *H*. *pluvialis*, and they share different molecular mechanisms of SA improving the astaxanthin synthesis. It can also be confirmed that although the biomass is increased slightly by SA-treatment, however, its degree is far less than those of astaxanthin content (the ratio of astaxanthin to biomass) and yield (mg/L). This result showed that the increasement in astaxanthin content is caused by the increased astaxanthin accumulation by SA-treatment, but not by increased biomass, while the increasement in astaxanthin yield is the combined result of increased biomass and astaxanthin biosynthesis.

Through transcriptomic technology, we compared the transcriptomic profiles of the samples under the SA-treatment and SA-free conditions (the S and C group). It was found that SA treatment could distinguish well the transcriptomic profiles of *P. rhodozyma* cells from the two groups, which was consistent with promotional effects of SA on biomass and astaxanthin accumulation. Further differentially expressed genes analysis (DEGs), which can explain variation in transcriptomic profile induced by SA treatment, was performed. Surprisingly, the regulatory range of SA on *P. rhodozyma* cells are far beyond our expectations. A metabolic network, involved in astaxanthin biosynthesis, central carbon metabolism, fatty acid metabolism, sterol metabolism, anti-stress, biomolecules transportation, DNA metabolism and signal transduction were regulated by SA treatment, which suggested that the regulatory mechanism of SA was very complex and explained why the *P. rhodozyma* was so sensitive to the SA phytohormone.

Phytohormones are considered to have a dual function in plant and microorganism. 1) The regulation of biomass and primary metabolite accumulation. 2) The response to abiotic stresses, allowing different species to adapt to the prevailing conditions (Stirk and van Staden, 2020). In the astaxanthin synthesis pathway, only astaxanthin synthase was identified as an upregulated DEG with the other genes in this pathway not significantly changed. In addition, pyruvate kinase and isocitrate dehydrogenase which produce ATP and NADPH and provide the energy and reducing power for astaxanthin synthesis, were induced by SA treatment. On the other hand, the fatty acid and sterol metabolisms are the competitive pathways of the astaxanthin synthesis and share the same precursor Acetyl-CoA and reducing power NADPH with astaxanthin synthesis. SA treatment inhibited the fatty acid and sterol synthesis, while improved the fatty acid degradation, which enhanced the astaxanthin synthesis metabolic flux and provided it with more precursors. In *H. pluvialis*, the effects of SA were found to be more pathway-specific, and 25 and 50 mg/L SA could enhance transcriptional expressions of eight carotenoid genes and seven astaxanthin biosynthesis genes, such as ipi-1, ipi-2, psy, crtR-B, bkt, crtO and pds. *The effects of SA on the other pathways have been less reported (* Gao et al., 2012 *)*.

*Regulation of SA on nucleotide metabolism has been reported only in higher plant. For example, SA was found to enhance the activity of dihydrofolate reductase–thymidylate synthase (DHFR–TS) and DNA methylation in Moringa oleifera Lam. Leaves to stimulate its growth (* Saini et al., 2014 *). However, related research in microorganism have never been reported. In this study, we found that SA stimulated nucleotide synthesis through up-regulating 5 related enzymatic genes, resulting in faster replication of genetic material and higher biomass in P. rhodozyma*. Meanwhile, transportations of key molecules play important role in cellular physiological activity and growth. Induction of transporters for molecules, such as metal ions, amino acids, *nucleotides, oligopeptide, and so on,* could strengthen cellular metabolism and explain the higher biomass induced by SA.

In *P. rhodozyma*, 22°C as a low-temperature stress condition in this study, is beneficial for astaxanthin accumulation while inhibit cell growth, affecting astaxanthin yield (Li et al., 2008). Thus, the anti-stress mechanism induced by phytohormone is one of the research hotpots. In the anti-stress group of DGEs, glutathione S-transferase, catalase, peroxiredoxin and thioredoxin are ROS scavenging enzymes, and can protect cells from ROS damage (Du et al., 2021). Spermine/spermidine is a polyamine-type of antioxidant, and the inductions of their synthase and transporter by SA treatment in this study are also the antioxidation mechanism (Zhou et al., 2020). Spermine/spermidine are widely found in prokaryotic and eukaryotic cells and participate in plant developmental metabolism. It was found that the salt tolerance of apple calli and the heat tolerance of wheat could be improved after treatment with spermine/spermidine (Liu et al., 2006; Kumar et al., 2014). The regulation of plant development and metabolism was coordinated by integrating polyamines with phytohormone (Ke et al., 2018). The exogenous and endogenous polyamine could increase astaxanthin and lipid synthesis through inducing the expressions of carotenogenic, lipogenic and antioxidant enzyme genes (Xing et al., 2022). Gamma-aminobutyrate (GABA), an important signaling molecule, plays an important role in regulating metabolism and cell growth (Seifikalhor et al., 2019). In this study, GABA permease, involved in GABA transportation in cell, was induced significantly by SA, suggesting that the GABA signal transportation participated in the astaxanthin synthesis regulation. ABC transporters play an important role in the accumulation and transmembrane transport of secondary metabolites (Theodoulou and Kerr, 2015). Carotenoids, including astaxanthin, are generally stored in the cells and are not automatically released from the cells (Lv et al., 2016). Gibberellic acid (GA) was found to induce the ABC transporters in *P. rhodozyma* to enhance the astaxanthin production (Liu et al., 2022). In this study, we identified an ABC transporter of *P. rhodozyma* as an upregulated DEG induced by SA. In *Saccharomyces cerevisiae*, overexpression of ABC transporters could promote the secretion of carotene (Bu et al., 2020). These results indicated that ABC transporters in *P. rhodozyma* is a candidate efflux pumps and a target of transporter engineering for enforcing astaxanthin production.

SA is a signal molecule, thus the signal pathways induced by or interacted with this molecule are essential in regulation of astaxanthin synthesis by SA. In this study, we found that SA could induce some important signal pathways, including the MAPK signal pathway (srf-type Transcription factor, STE3 and SH01), Ca/calmodulin pathway (Ca/calmodulin-dependent protein kinase). Meanwhile, two global transcription factors (TFs), basic-leucine zipper and basic helix-loop-helix (bHLH), were also induced by SA, suggesting that these signal pathways play important roles in the global regulation of astaxanthin biosynthesis in *P. rhodozyma*. The mitogen-activated protein kinases (MAPKs) are a key conserved signaling pathway used to transduce extracellular induces into intracellular responses in eukaryotes (Moustafa et al., 2014). In *H. pluvialis*, it was found that the expressions of MAPK pathway genes were positively correlated with astaxanthin accumulation, and *vice versa*. Moreover, MAPK pathway was a target of NO action in *H. pluvialis* (Zhao et al., 2019b). In higher plant, MAPK pathway can modulate the other signal pathways, such as ROS and ABA pathways, to responses to various stresses (Raja et al., 2017). Ca/Calmodulin (CaM) is a signal molecule and can activate glutamate decarboxylase (GAD) which catalyzes γ-Aminobutyric acid (GABA) synthesis. A salt stress treatment could induce the transcription of CaM to enhance GABA accumulation in *H. pluvialis* (Ding et al., 2019). Moreover, CaM participate indirectly in managing ROS levels through CaM-regulated GABA synthesis and the GABA shunt metabolic pathway (Bouché et al., 2003). The results derived from our current study was consistent with the previous studies, and we deduced that interactions of various signal molecules or pathways, such as SA, MAPK, ROS, GABA, co-regulated the astaxanthin synthesis in *P. rhodozyma*.

**FIGURE 12:**
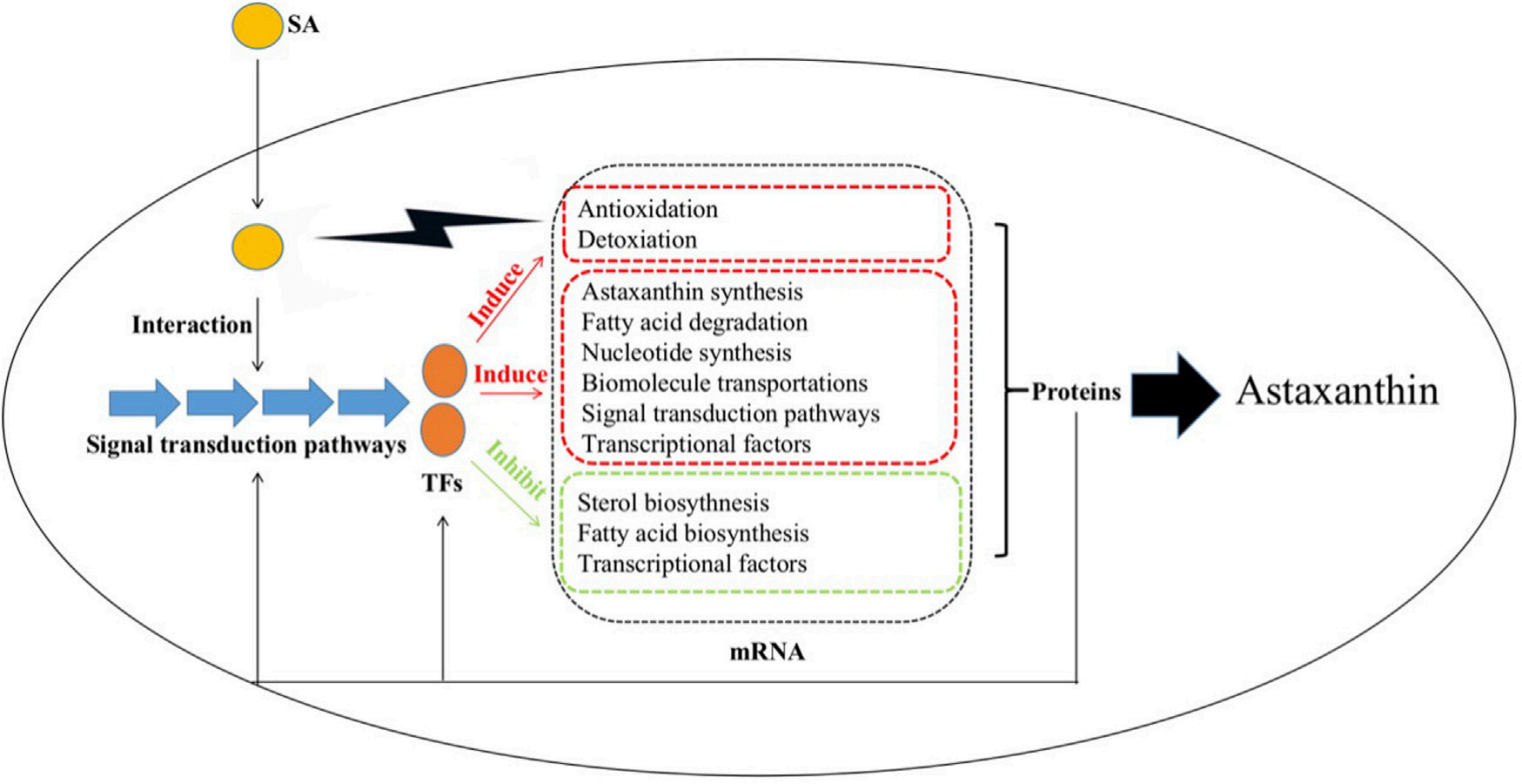
Schematic diagram for the molecular mechanism of SA improving astaxanthin synthesis in *P*. *rhodozyma* at the global-cell level SA enters yeast cells and interacts with other signaling pathways to transmit the signals to the transcription factors (TFs), which can induce the transcriptions of genes related to antioxidation, detoxication, astaxanthin biosynthesis, fatty acid degradation, nucleotide biosynthesis, biomolecules transportations, TFs, signal pathways, while can inhibit the transcriptions of genes related to sterol and fatty acid biosynthesis, and TFs, leading to changes in their protein products. The proteins in turn affect related signaling pathways and TFs and enhance the astaxanthin production.

Based on the results from the current study, we propose the global regulation mechanism of SA improving astaxanthin synthesis in *P. rhodozyma* (Figure 12). SA is first perceived by *P. rhodozyma* cell and enters cells. As a signal molecule, SA can interact with the MAPK and Ca/calmodulin signal pathways and regulate their signal transductions. The regulation signals are transduced to the transcription factors (TFs), such as srf-type, STE3, SH01, basic-leucine zipper and basic helix-loop-helix TFs. The signalized TFs enter nucleus and regulate the expressions of various downstream genes related to astaxanthin and its competitive metabolites synthesis, cell growth, anti-stress and detoxication, signal pathways and TFs. In addition, SA can exert oxidative or toxic stress on the *P. rhodozyma* cells, prompting them to produce more astaxanthin for antioxidation and detoxification.

Based on the transcriptomic data induced by SA, overexpression of the upregulated DEGs or knock-out of the downregulated DEGs in *P. rhodozyma* is a potential strategy to further improve astaxanthin productivity, and the identified DEGs are potential targets for genetic engineering in this astaxanthin-producing yeast. In this study, a novel polyamine transporter gene (PT), an upregulated DEG induced by SA, was selected to be overexpressed in *P. rhodozyma*. A transformant Prh-PT-006 with the PT gene overexpressed in *P. rhodozyma* was obtained and its astaxanthin content and yield were further increased compared to the WT strain under SA-treatment condition. In microorganism, polyamine (putrescine, spermidine and spermine) is closely related to the resistance to biotic or abiotic stresses. Polyamines are essential for cell division (Schneider and Wendisch, 2011), and bind anionic structures such as plasma membranes, ribosomes, and DNA, to protects DNA from damage by some reactive compounds, regulates gene expression, and modulates translational fidelity (Tkachenko et al., 2001). On the contrary, excess polyamines are toxic, displacing magnesium from ribosomes and inhibiting protein synthesis (Limsuwun and Jones, 2000). Concentrations of polyamines are maintained within a specific range and intracellular levels of polyamines are regulated by expression of the polyamine transporter gene. Thus, polyamines transporters are essential to maintain its homeostasis. In *E. coli*, polyamines protect DNA from damage by agents of oxidative stress (Tkachenko et al., 2012). Furfural is a fermentation inhibitor, and overexpression of polyamine transporter gene increase furfural tolerance to improve xylose fermentation in *E. coli* (Geddes et al., 2014). In pathogenic yeast *C. albicans*, a polyamine efflux transporter Flu1 can efflux the fungicide Histatin 5 (Hst 5) to reduce cytosolic levels of Hst 5. Overexpression of *Flu1* can significantly reduce Hst 5’s toxicity in *Candida albicans* (Li et al., 2013). Thus, the mechanism in this study may be that the stress condition, such as low temperature, is benefit for astaxanthin accumulation, but is harmful for cell growth. Overexpression of the PT gene can increase the intracellular levels of polyamines, which enhances the cell’s anti-stress ability to maintain cell’s normal growth. Astaxanthin is a super antioxidant and increase in its productivity by overexpression of the PT gene is also a cellular anti-stress mechanism.

## 5 Conclusion

An extremely low concentration of exogenous SA could significantly promote cell growth and astaxanthin accumulation in *P. rhodozyma* and could completely distinguish the transcriptomic profile of cells from the SA-free sample. Thus, *P. rhodozyma* is very sensitive to the phytohormone SA, and SA is a potential strategy to break through the bottleneck of microbial astaxanthin production. SA regulates astaxanthin synthesis in *P. rhodozyma* at global-cell level, which is involved in astaxanthin and its competitive metabolite synthesis, material supply, biomolecules metabolism and transportation, anti-stress, and signal transductions. The regulation mechanism of SA on astaxanthin synthesis in *P. rhodozyma*, including the perception and transduction of SA signal, the transcription factor, the expression regulations of the downstream genes, and cellular stress response, is put forward for the first time. Moreover, overexpression of an upregulated DEG, polyamine transporter (*PT*) gene, can further improve astaxanthin productivity in *P. rhodozyma*. The biomass, astaxanthin content and yield in the transformant overexpressing PT gene under SA-treatment condition are significantly increased compared to the WT under SA-free condition. This study presents a combined strategy of SA-treatment and PT overexpression for astaxanthin production, and our results also provide the targets of genetic modification for constructing microbial strains with high-yield of astaxanthin.

## Data Availability

The data presented in the study are deposited in the Gene Expression Omnibus (GEO) repository of National Center for Biotechnology information (NCBI), accession number GSE242572.
